# Effect of Metformin on the Prognosis of Gastric Cancer Patients with Type 2 Diabetes Mellitus: A Meta-Analysis Based on Retrospective Cohort Studies

**DOI:** 10.1155/2023/5892731

**Published:** 2023-03-04

**Authors:** Lingna Li, Jianing Huang, Tongmin Huang, Jie Yao, Yeyuan Zhang, Meiling Chen, Haojie Shentu, Haiying Lou

**Affiliations:** ^1^Pharmacy Department, The Affiliated Hospital of Ningbo University, Li Huili Hospital, Ningbo, China; ^2^School of Public Health, Zhejiang Chinese Medical University, Hangzhou, Zhejiang, China; ^3^The Second Clinical Medical College, Zhejiang Chinese Medical University, Hangzhou, Zhejiang, China; ^4^School of Medical Imaging, Hangzhou Medical College, Hangzhou, Zhejiang, China; ^5^Department of Endocrinology, Zhuji People's Hospital, Shaoxing, Zhejiang, China

## Abstract

**Background:**

Metformin is one of the most common drugs for type 2 diabetes mellitus (T2DM) treatment. In addition, metformin intends to have a positive effect on the prognosis of several cancers. However, the therapeutic effect of metformin on gastric cancer (GC) remains controversial. This study explores and updates the therapeutic effect of metformin in GC patients with T2DM.

**Methods:**

We searched through PubMed, Embase, Web of Science, and the Cochrane Library for relevant articles by July 2022. The relationship between metformin therapy and the prognosis of GC patients with T2DM was evaluated based on the hazard ratio (HR) at a 95% confidence interval (95% CI). Overall survival (OS), cancer-specific survival (CSS), and progression-free survival (PFS) were the primary outcomes analyzed.

**Results:**

Seven retrospective cohort studies with a combined 2,858 patients met the inclusion criteria. OS and CSS were reported in six studies, and PFS was reported in four studies. Pooled results showed that, compared to the nonmetformin group, the prolonged OS (HR = 0.72, *p* = 0.001), CSS (HR = 0.81, *p* = 0.001), and PFS (HR = 0.70, *p* = 0.008) of the experimental group may be associated with the exposure to metformin.

**Conclusion:**

Metformin may have a beneficial effect on the prognosis of GC patients with T2DM.

## 1. Introduction

Gastric cancer (GC) is one of the most common cancers and the fourth leading cause of cancer-correlated death in the world [1, 2]. Every year, approximately 990,000 individuals are diagnosed with GC, resulting in 738,000 deaths. Central and South America, Eastern Europe, and East Asia have the highest incidences of GC [3]. In addition to genetic and dietary factors, helicobacter pylori (H. pylori), a Gram-negative bacteria promotes the development of GC [4]. Surgery combined with adjuvant chemotherapy is the only potentially curative treatment for GC [5, 6]. However, the 5-year-related survival rate of GC patients after surgery is between 10% and 30% [7]. Consequently, there is an urgent need to explore and develop other universal and effective therapies for GC treatment.

Type 2 diabetes mellitus (T2DM) is the 8^th^ most prevalent disease globally, and the prevalence rate increased from 2.88% in 1990 to 5.89% in 2019 worldwide [8]. A recent review estimated that in 2012, about 293,000 cancer cases globally were attributed to T2DM [9]. Accordingly, the treatment of T2DM might be beneficial for improving the prognosis and relieving the burden of cancer [10]. Among them, metformin, a first-line pharmacologic treatment for T2DM, poses anticancer properties in an article which formally proposed that metformin could impair the metabolic plasticity and growth of tumor with the combination of hypoglycemia by modulating the PP2A-GSK3*β*-MCL-1 Axis at the molecular biochemical level [11–14]. Studies have shown that metformin has a favorable effect on the prognosis of several cancers including lung cancer [15–17], prostate cancer [18, 19], colorectal cancer [20, 21], breast cancer [19, 22–24], and endometrial cancer [25].

Nevertheless, whether metformin could improve the prognosis of GC patients with T2DM still remains controversial. A study claimed that metformin decreases the recurrence, all-cause mortality, and cancer-specific mortality rates of GC patients with diabetes after gastrectomy, more significantly with the increase of dose [26]. Another study stated that the use of metformin improves the overall mortality of GC patients with T2DM, but no association was found in a cancer-specific survival (CSS) [27]. A recent study revealed that metformin does not improve the prognosis of GC patients with T2DM [28]. Herein, the aim of our study is to render an update on the new data on the therapeutic value of metformin on the prognosis of GC patients with T2DM by exploring and updating correlative research systematically.

## 2. Materials and Methods

### 2.1. Search Strategy

This meta-analysis was conducted in accordance with the principles of Preferred Reporting Items for Systematic Review and Meta-analyses (PRISMA) [29]. We systematically searched through PubMed, EMBASE, Web of Science, Medline, and the Cochrane Library to identify clinical articles from inception to July 2022 on the therapeutic effect of metformin on the prognosis of GC patients with T2DM. The search terms were as follows: (“Neoplasm, Stomach” OR “Stomach Neoplasm” OR “Neoplasms, Stomach” OR “Stomach Neoplasms” OR “Gastric Neoplasms” [MeSH Terms] OR “Gastric Neoplasm” OR “Neoplasm, Gastric” OR “Neoplasm, Gastric” OR “cancer of stomach” OR “Stomach cancers” OR “Gastric cancer” OR “cancer, gastric” OR “cancers, gastric” OR “gastric cancers” OR “Stomach cancer” OR “Cancer, Stomach” OR “Cancers, Stomach” OR “Cancer of the Stomach” OR “Gastric Cancer, Familial Diffuse”) and (“Metformin”) [MeSH Terms] OR “Dimethylbiguanidine” OR “Glucophage” OR “Metformin Hydrochloride” OR “Hydrochloride, Metformin” OR “Metformin HCL” OR “HCL, Metformin”) and (“Prognosis” [MeSH Terms] OR “Prognoses” OR “Prognostic Factors” OR “Prognostic Factor” OR “Factor, Prognostic” OR “Factors, Prognostic” OR “Comes” OR “outcome” OR “outcomes” OR “Survival” OR “Mortality” OR “Overall Survival” OR “OS” OR “objective response rate” OR “ORR” OR “progression-free survival” OR “PFS” OR “time to progress” OR “TTP” OR “disease-free survival” OR “DFS” OR “Recurrence-free survival” OR “RFS”). Studies were searched primarily in English. Relevant articles in references of the selected studies were retrieved as well to avoid omissions. The searches were conducted independently by two investigators. Divergences would be tackled at the discretion of a third investigator.

### 2.2. Inclusion and Exclusion Criteria

Inclusion of the included articles was based on the PICOS criterion: (1) Population: GC patients with T2DM; (2) Intervention: exposure to metformin; (3) Control: nonexposure to metformin (other antidiabetic medicines, treatment, or placebo); (4) Outcome: cancer survival indicators; (5) Studies: cohort studies, case-control studies, and cross-sectional studies.

The surplus selection criterion was defined as follows: (1) observational studies on the prognostic effect of metformin on GC patients with T2DM; (2) exposure to metformin for patients in the experimental group and nonmetformin for patients in the control group; (3) available tumor-related survival data. If the data from different publications is from the same participation, priority will be given to the most recent or systematic one.

Exclusion criterion was set as below: (1) studies without full text; (2) data results that cannot be counted; (3) protocols without specific results; (4) studies that explored multiple factors but lacked interesting outcomes.

The observational outcome indicators of prognosis included in this study were overall survival (OS), progression-free survival (PFS), and CSS.

### 2.3. Quality Assessment and Data Extraction

The quality of the selected studies was evaluated using the Newcastle-Ottawa Scale (NOS), which consists of three components: selection, comparability, and outcome. Selection included four parts: representativeness of the exposed cohort; selection of the nonexposed cohort; ascertainment of exposure; and outcomes not present at the start of the study. Outcomes included three items: assessment of outcomes; length of follow-up; and adequacy of follow-up. Studies with a total evaluation score ≥7 were of high quality [30].

Data extracted from the selected articles included author, year of publication, country of research, sample size, sex, age, body mass index (BMI) of the study participants, tumor node metastasis stage (TNM stage), metformin therapeutic scheme and mode of administration, interventions in the control group, GC therapy, and type of gastrectomy. Any disagreement between the two investigators was resolved through arbitration by a third investigator.

### 2.4. Statistical Analysis

Data analysis was performed using Stata software, version 12.0. The association between metformin therapy and the prognosis of GC patients with T2DM was analyzed based on the hazard ratio (HR) at a 95% confidence interval (95% CI) by utilizing a random-effects model to increase reliability, giving consideration to potential heterogeneity between studies: the difference in the dosage of treatment received, differences at baseline, inconsistent GC staging, and nonuniform treatment of the control groups. Heterogeneity was evaluated using Chi-square test in order to determine if there was a significant difference in the selected data. *I*^2^ < 50% indicated low heterogeneity, while *I*^2^ ≥ 50% indicated high heterogeneity [31]. If the number of articles included is more than ten, sensitivity analysis was performed to examine the stability of the pooled results, and Begg's regression test was conducted to examine the publication bias of the results. *P* value less than 0.05 indicated a statistically significant difference.

## 3. Results

### 3.1. Search Results

**Figure 1:**
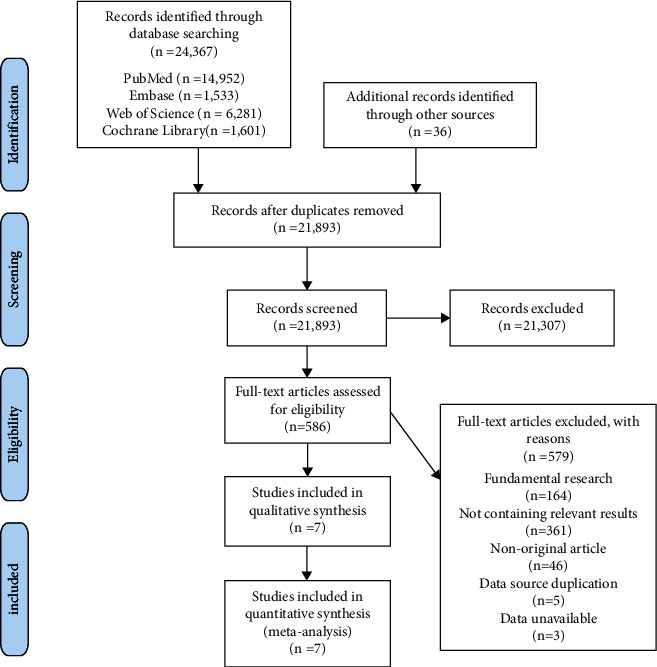
Flow diagram for the selection process of included studies.

The initial literature search from the online databases revealed 24,367 studies. The additional searches of the reference materials in the retrieved studies generated 36 studies. After removing duplicates, 21,893 studies were retained. After reading the titles and abstracts and removing irrelevant articles, we read the full text of 586 studies for potential inclusion. Of these articles, 579 articles were further excluded: 164 were fundamental research on molecular, biochemical, or animal studies; 361 did not capture relevant outcomes; 46 were nonoriginal articles; five were duplicates; and three had unavailable data. A total of seven studies [26, 27, 32–36] were included for quantitative analysis. The flowchart of the entire selection process is presented in Figure 1.

### 3.2. Study Characteristics

Table 1 shows the basic characteristics of the seven included studies included in our meta-analysis. Two studies were conducted in China, two in Korea, and one each in America, Belgium, and Sweden. All the included articles were cohort studies published in the last six years involving a total of 2,858 patients. The metformin exposure group consisted of 1,620 patients, while the control group contained 1,238 patients. A total of 1,141 patients had stage I-II tumors, while 1,134 patients had stage III-IV tumors. All the studies adjust for factors including but not limited to sex, age, BMI, TNM stages, and gastrectomy. Detailed data on the study population are shown in Supplementary Table 1.

### 3.3. Quality Assessment

The quality assessment for the selected studies was evaluated using NOS. All the studies had NOS scores ranging from 7 to 9, indicating high quality and low risk of bias. A specific assessment with perspective aspects is shown in Supplementary Table 2.

### 3.4. Prognostic Analysis

**Figure 2:**
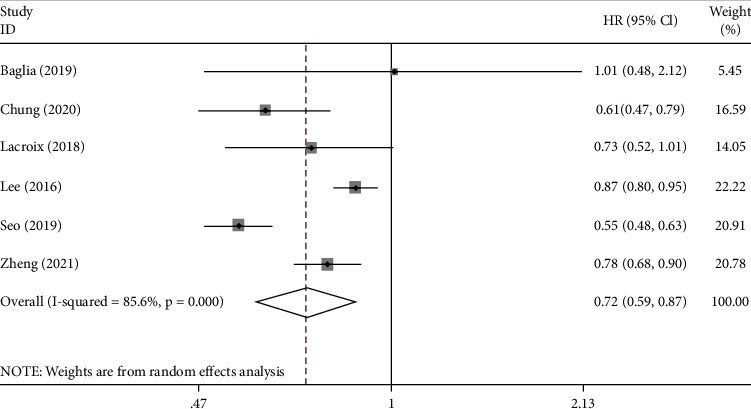
Forest plot of the overall survival (OS) of GC patients with T2DM after exposure to metformin (*p* = 0.001).

Six studies reported the HRs of the relationship between metformin and the OS of the GC patients with T2DM. Pooled results reveal that compared with the control group, the OS rate of the metformin exposure group was significantly high (HR = 0.72, 95% CI: 0.59–0.87, *p* = 0.001). The heterogeneity of the pooled results was significant (*I*^2^ = 85.6%). The detailed results are shown in Figure 2.

**Figure 3:**
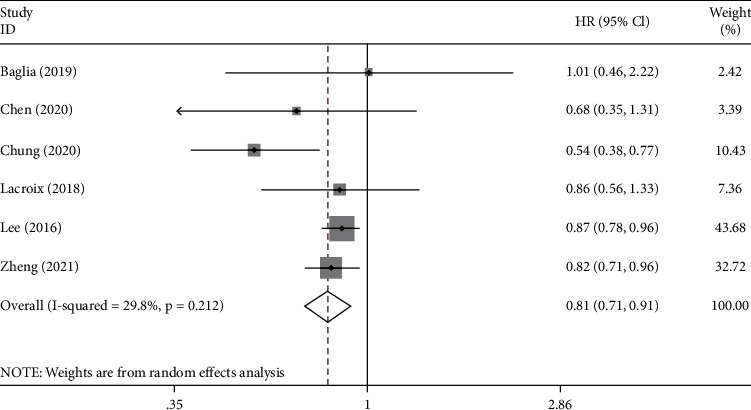
Forest plot of the cancer-specific survival (CSS) of GC patients with T2DM after exposure to metformin (*p* = 0.001).

Six studies reported the relationship between metformin and CSS in GC patients with T2DM. The pooled results revealed that the CSS rate was higher in the metformin exposure group than in the control group (HR = 0.81, 95% CI: 0.71–0.91, *p* = 0.001). The heterogeneity among the studies was very low (*I*^2^ = 29.8%). The details are shown in Figure 3.

**Figure 4:**
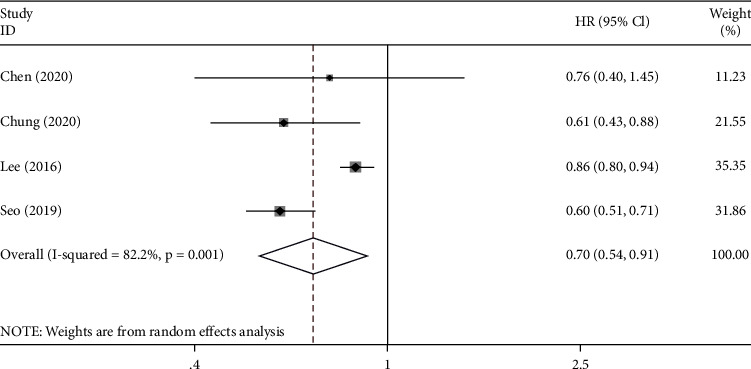
Forest plot of the progression-free survival (PFS) of GC patients with T2DM after exposure to metformin (*p* = 0.008).

Four studies reported on the PFS. Pooled analysis indicated that metformin increased the PFS of GC patients with T2DM compared with the control group (HR = 0.70, 95% CI: 0.54–0.91, *p* = 0.008). However, the heterogeneity among the studies was very high (*I*^2^ = 82.2%) (Figure 4).

## 4. Discussion

Metformin, a first-line treatment for T2DM, has been considered to have an improved effect on the prognosis of cancer patients. However, before this study, the role of metformin in GC remained controversial. This study summarized the current data on the effect of metformin on the prognosis of GC patients with T2DM. Our study showed that metformin might be beneficial for the OS of GC patients with T2DM. Since all the included patients had T2DM, the prognostic effect of metformin may stem from the treatment of diabetes. CSS was further analyzed to rule out this potential factor, implying that metformin may have a positive effect on the CSS of GC patients with T2DM. Additionally, we found that metformin might contribute to a longer PFS in GC patients with T2DM.

To explore mechanisms for the influence of metformin on GC, potential causes were listed as follows:

First, T2DM promotes GC progression and related mortality [33]. Therefore, treating T2DM may improve GC prognosis. In T2DM patients, the hyperglycaemic environment provides sufficient energy for the growth and proliferation of GC cells and inhibits apoptosis [37]. A common complication of diabetes mellitus is nerve injury, and persistent hyperglycemia will lead to neuronal damage and promote the perineural metastasis of cancer cells [38]. So metformin reduces hyperglycaemic to limit the proliferation and growth of GC cells by inhibiting hepatic gluconeogenesis and stimulating peripheral glucose uptake. Otherwise, metformin lowers insulin levels in GC patients. This inhibits insulin binding to the insulin receptor (IR) [39]and refrains from activating the insulin-like growth factor (IGF) receptor signalling axis [40, 41], therefore inhibiting tumor growth.

Second, from the cellular metabolism perspective, metformin inhibits the development of GC cells by inhibiting cellular autophagy, glutaminase activity, mitochondrial function, nicotinamide adenine dinucleotide (NADH) dehydrogenase, and inducing phosphorylation of epigenetic enzymes.

1. Metformin inhibits cellular autophagy that supports tumor survival and growth by reducing GC intracellular metabolism in the terminal stage [42, 43] and protects against hypoxia and nutrient deficiency [44].
2. Metformin impairs glutaminase activity [45] and reduces the proliferation of GC cells. A key metabolic characteristic of many cancer cells is their high dependence on glutamine.
3. Metformin impairs mitochondrial activity, inhibits cellular respiration, and subsequently induces GC cell death [46].
4. Under hypoglycemic conditions, metformin strongly induces cell death by inhibiting the NADH dehydrogenase. This breaks the electron transport chain, and the anticancer effect may be greatly enhanced [47].
5. Metformin phosphorylates and inhibits the function of several epigenetic enzymes, such as histone acetyltransferases (HATs), class II histone deacetylases (HDACs), and DNA methyltransferases (DNMT). Epigenomic modifications of metformin enhance its anticancer properties [48].

Third, immunologically, metformin exerts antitumor activities by increasing CD8+ T cells [49, 50], inhibiting apoptosis of CD8+ tumor-infiltrating lymphocytes [49, 51], and neutralizing immune-inhibitory cell populations in the tumor microenvironment [52] to realize effective cancer immunotherapy. Additionally, metformin slows the rate of GC cell transformation rate by inhibiting mediators of the inflammatory response, including transcription factors and inflammatory molecules [53].

More importantly, in terms of molecular pathways, adenosine 5′-monophosphate (AMP)-activated protein kinase (AMPK) is a crucial pathway that regulates the development of GC cells. The AMPK pathway is activated in two ways. First, metformin directly activates AMPK, thereby inhibiting the downstream Akt/mTOR signalling pathway and resulting in the generation of cancer cells [54–56]. Therefore, inhibition of the PI3K/Akt/mTOR pathway disrupts cancer cell proliferation [57, 58]. Metformin also activates AMPK by inhibiting and impairing the function of mitochondrial complex I [59] and inhibits cellular cell respiration and later triggers GC cell death [46].

Activation of AMPK triggers a series of reactions: (1) the expression of p53, an antioncogene in humans [60]; (2) inhibition of the expression of the fatty acid synthase (FAS) gene, modulating lipogenesis and inhibiting proliferation [57, 61], stemness, and chemoresistance [62] of GC cell; and (3) regulation of cyclin D1 and the cyclin-dependent protein kinases p21 and p27, which contribute to their anticancer effects [63, 64]. The antitumor effects of metformin on GC and peritoneal metastases in vitro relies on pathways related to the AMPK pathway [65].

This study presented that metformin may improve the prognosis of GC patients through the above mechanisms. Although one study reported no association between the use of metformin and GC mortality in GC patients [66], metformin tended to exert a positive effect at different stages of GC. At the stage of gastric disease, metformin can slow down the progression of gastric disease due to H. pylori infection, suggesting that metformin may be beneficial for early gastric disease stage [67]. During the chemotherapy period for GC, metformin, in combination with oxaliplatin could inhibit the proliferation of GC cells and further induce their apoptosis [68]. In a GC cell line model, metformin in coordination with curcumin increased the cytotoxic effects of anticancer drugs on GC cells [69]. Metformin can also replace specific small interfering RNA (siRNA) to inhibit the migration of human gastric adenocarcinoma (AGS) cell lines, a type of GC cells [70]. Generally, numerous studies showed positive results and demonstrated the beneficial effect of metformin on GC patients.

This article is an updated meta-analysis on the prognostic impact of metformin on GC patients with T2DM. The results of our study, including three prognostic indicators—OS, CSS, and PFS, are all statistically significant. However, this study has some limitations as well.

First, the pooled results of OS and PFS are of high heterogeneity. As for the reasons, we have considered the following aspects:

1. In terms of exposure time and dose of metformin, the specific dose and length of exposure time in the experimental group may have influenced the results. Unfortunately, some articles did not mention the dose of metformin given to the experimental group, and two articles that studied the dose relationship had different dose classifications and exposure time was not mentioned, so we could not conduct an in-depth study.
2. In aspects related to blood glucose, the control of blood glucose by metformin may affect the prognosis of patients. Metformin may have a better effect on controlling the glycemic situation better than other hypoglycemic drugs. Unfortunately, due to the insufficient number of articles and the lack of relevant data, we cannot further study the relationship between the prognosis of gastric cancer patients and the blood glucose of patients, and we cannot exclude the possibility that it is because the metformin group had probably a shorter diabetes duration. If available, insulin resistance and the administration of other hypoglycemic drugs were also supposed to be analyzed.
3. Eternal time bias may also explain the source of heterogeneity. For patients assigned to the experimental group, if gastric cancer progression or even death occurred before taking metformin, their progression and mortality from gastric cancer would be underestimated, and the prognostic effectiveness of metformin would be overestimated. Therefore, studies need to be designed to ensure that the three time points (follow-up initiation, compliance with inclusion criteria, and exposure allocation) are uniform. Unfortunately, the seven included cohort studies did not provide sufficient data for us to calculate the magnitude of the immortal time bias, so we could not know the specific impact of the immortal time bias on the pooled results. Therefore, we used a random-effects model to combine effect sizes in the analysis of the data to improve the precision of the estimated confidence intervals and increase the power of the test.

Secondly, in perspective of study regions, three of these seven included articles are from China, two are from Korea, and two are from Europe. So the pooled results tend to show that metformin may improve OS, CSS, and PFS in Asian populations, and more studies are still needed to verify the PFS in European populations.

## 5. Conclusion

The pooled results of this meta-analysis showed that metformin may have a positive effect on the prognosis of GC patients with T2DM. However, further multicenter studies of larger-scale clinical trials are still needed to validate the findings of this meta-analysis.

## Figures and Tables

**Table 1:** Characteristics of all the studies included in the meta-analysis.

Authors	Years	Countries	Sample size	Met users	Nonmet users	TNM stage					Adjusting factors
						I	II	III	IV	Unknown	
Baglia	2019	American	130	81	49	NA					Sex, education, BMI, smoking, exercise, comorbidities, TNM stages, and cancer treatment
Chen	2020	China	71	51	20	16	4	3	48	0	Sex, age, hepatitis, tumor location, gastrectomy, reconstruction, lymphadenectomy, radical resection, and TNM stages
Chung	2020	China	651	248	403	239	137	275	0	0	Sex, age, Charlson comorbidity index, gastrectomy, lymphadenectomy, tumour size, differentiation, resection margins, TNM stages, vascular invasion, lymphatic invasion, perineural invasion, chemotherapy, and hypoglycemic medications
Lacroix	2018	Belgian	298	228	70	129	87	82	0	0	Age, sex, year of diagnosis, TNM stages, cancer treatment, and comorbidities
Lee	2016	Korea	326	132	194	178	60	88	0	0	Sex, age, BMI, use of insulin, cancer treatment, and TNM stages
Seo	2019	Korea	242	103	139	NA					Age, sex, ECOG, gastrectomy, and TNM stages
Zheng	2021	Sweden	1140	777	363	291		638		211	Sex, age, year of diagnosis, use of nonsteroidal anti-inflammatory drugs or aspirin, use of statins, and Charlson comorbidity index

Met, metformin; Nonmet, nonmetformin; NA, not available; TNM, tumor node metastasis classification; BMI, body mass index; ECOG, eastern cooperative oncology group.

## Data Availability

The data supporting the findings reported in this study are available in the supplementary materials.

## Abbreviations

GC: Gastric cancer
H. pylori: Helicobacter pylori
T2DM: Type 2 diabetes mellitus
CSS: Cancer-specific survival
PRISMA: Preferred reporting items for systematic review and meta-analyses
OS: Overall survival
PFS: Progression-free survival
NOS: Newcastle-Ottawa scale
BMI: Body mass index
TNM stage: Tumor node metastasis stage
HR: Hazard ratio
95% CI: 95% confidence interval
ATP: Adenosine triphosphate
IR: Insulin receptor
IGF: Insulin-like growth factor
TCA: Tricarboxylic acid
AMP: Adenosine 5′-monophosphate
AMPK: Adenosine 5′-monophosphate (AMP)-activated protein kinase
FAS: Fatty acid synthase
NADH: Nicotinamide adenine dinucleotide
HATs: Histone acetyltransferase
HDACs: Class II histone deacetylases
DNMT: DNA methyltransferases
si RNA: Small interfering
AGS: Human gastric adenocarcinoma.

## Supplementary Materials

Supplementary Materials Supplementary Table 1. Supplementary characteristics of studies included in the meta-analysis. Supplementary Table 2. Quality assessment of studies included. PRISMA 2009 checklist. Preferred Reporting Items for Systematic Reviews and Meta-Analyses: The PRISMA Statement.
