# Supplementary material for: Effect of Metformin on the Prognosis of Gastric Cancer Patients with Type 2 Diabetes Mellitus: A Meta-Analysis Based on Retrospective Cohort Studies
Source: Int J Endocrinol. 2023 Mar 4;2023:5892731. doi: 10.1155/2023/5892731 (PMC10008112; doi:10.1155/2023/5892731)
Supplement: Supplementary Materials — Supplementary Table 1. Supplementary characteristics of studies included in the meta-analysis. Supplementary Table 2. Quality assessment of studies included. PRISMA 2009 checklist. Preferred Reporting Items for Systematic Reviews and Meta-Analyses: The PRISMA Statement. [file 5892731.f1.zip › Supplementary Table 1.docx]

**Supplementary Table 1. Characteristics of all the studies included in the meta-analysis.**

| Author | Year | Source of exposure data | Period | Sex | | Age | Gastrectomy | | |
| --- | --- | --- | --- | --- | --- | --- | --- | --- | --- |
|  |  |  |  | female | male |  | Total | Partial | Others |
| Baglia | 2019 | participants of two population-based cohort studies in Shanghai | 2004-2015 | NA | NA | 40-70 | NA | | |
| Chen | 2020 | Nanfang Hospital, Southern  Medical University | 2004-2019 | 51 | 20 | ≤69 year: 57 cases  ≥70 year: 14 cases | 24 | 47 | 0 |
| Chung | 2020 | Chang Gung Memorial Hospital at Linkou | 1997-2016 | 392 | 259 | 67.35±10.76 | 159 | 492 | 0 |
| Lacroix | 2018 | the BCR database (International  Classifification of Diseases (ICD), 10th revision: C16.1–C16.9) | 2006-2015 | 169 | 129 | ≤69 year: 80 cases  ≥70 year: 148 cases | 154 | | 125 |
| Lee | 2016 | the Yonsei Cancer Center, Seoul, Republic of Korea | 2003-2010 | 247 | 79 | 59 (22–89) | 110 | | 216 |
| Seo | 2019 | Seoul St. Mary’s Hospital | 1989-2016 | 181 | 61 | 62.9±10.3 | 78 | 164 | 0 |
| Zheng | 2021 | a Swedish nationwide  population-based cohort, entitled the Swedish Prescribed Drugs  and Health Cohort (SPREDH) | 2005-2020 | 807 | 333 | 73 (66-80) | 297 | | 843 |

NA, not available
