# Supplementary material for: Effect of Metformin on the Prognosis of Gastric Cancer Patients with Type 2 Diabetes Mellitus: A Meta-Analysis Based on Retrospective Cohort Studies
Source: Int J Endocrinol. 2023 Mar 4;2023:5892731. doi: 10.1155/2023/5892731 (PMC10008112; doi:10.1155/2023/5892731)
Supplement: Supplementary Materials — Supplementary Table 1. Supplementary characteristics of studies included in the meta-analysis. Supplementary Table 2. Quality assessment of studies included. PRISMA 2009 checklist. Preferred Reporting Items for Systematic Reviews and Meta-Analyses: The PRISMA Statement. [file 5892731.f1.zip › Supplementary Table 2.doc]

**Supplementary Table 2.** Quality assessment of studies included.

| Author, year,  Study (Cohort study) | **Selection (Out of 4)** | | | | **Comparability**  **(Out of 2)** | **Outcomes(Out of 3)** | | | **Total**  **(Out of 9)** |
| --- | --- | --- | --- | --- | --- | --- | --- | --- | --- |
|  | Representativeness of exposed cohort | Selection of non-exposed cohort | Ascertainment  of exposure | Outcome not present at the start of the study |  | Assessment of outcomes | Length of follow-up | Adequacy of follow-up |  |
| Baglia, 2019 | 1 | 1 | 1 | 1 | 1 | 1 | 1 | 0 | 7 |
| Chen, 2020 | 1 | 1 | 1 | 1 | 2 | 1 | 1 | 1 | 9 |
| Chung, 2020 | 1 | 1 | 1 | 1 | 2 | 1 | 1 | 0 | 8 |
| Lacroix, 2018 | 1 | 1 | 1 | 1 | 2 | 1 | 0 | 0 | 7 |
| Lee, 2016 | 1 | 1 | 1 | 1 | 2 | 1 | 0 | 1 | 8 |
| Seo, 2019 | 1 | 1 | 1 | 1 | 2 | 1 | 1 | 1 | 9 |
| Zheng, 2021 | 1 | 1 | 1 | 1 | 2 | 1 | 1 | 0 | 8 |
